# Avulsion of the Femoral Attachment of the Medial Collateral Ligament of the Knee Associated to Complete Tear of the Posterior Cruciate Ligament: A Case Report

**DOI:** 10.1055/s-0037-1599256

**Published:** 2017-03-20

**Authors:** Sleiman Haddad, Andrea Sallent, Joan Minguell, Enric Castellet

**Affiliations:** 1 Department of Orthopaedics, Hospital Vall d'Hebron, Barcelona, Spain

**Keywords:** knee ligament injuries, medial collateral ligament avulsion, posterior cruciate ligament tear, combined ligament injuries, surgical repair

## Abstract

Medial collateral ligament (MCL) of the knee is one of the most commonly injured ligaments of the knee. Incidence of posterior cruciate ligament (PCL) injuries can vary widely. Conservative treatment has shown good clinical outcomes and relatively rapid return to play in both injuries alone. We present the case of a 38-year-old male who presented a combined MCL avulsion injury and PCL tear treated surgically. The PCL was reconstructed using the double-bundle Achilles allograft technique. Within the same surgery, a medial femoral incision was performed to reinsert the avulsion of the bone fragment rotated and distally retracted together with the MCL with bone anchors and Spike Washer. Two years after surgery, the patient enjoyed a 0/140-degree range of motion for flexion/extension. He had returned to sports and was pain-free. In conclusion, femoral avulsion of the MCL associated to PCL injury is a rare and nondescribed injury that, as opposed to most MCL isolated injuries, might benefit from early surgical reconstruction.

Medial collateral ligament (MCL) of the knee is one of the most commonly injured ligaments of the knee. 1 Most injuries result from a valgus stress, tibial external rotation, or a combination of both. The majority of injuries occur in young athletes during sports practice, especially skiing, ice hockey, and football. 2 MCL fibers are normally injured in the proximal third, with complete disruption being classified as a type III injury. While most acute injuries are treated orthopedically, surgical repair should be considered in cases of chronic instabilities and multiligamentous injuries. 2

Incidence of posterior cruciate ligament (PCL) injuries can vary widely. Its conservative treatment has shown good clinical outcomes and relatively rapid return to play. 3

We present the first case of a combined MCL avulsion injury and PCL tear in a skeletally mature athletic patient treated surgically. Through this report, we discuss the peculiarities of such injury and present the surgical outcomes.

## Case Report

A 38-year-old male presented to the emergency room unable to bear weight on his right knee after a fall while practicing recreational sport, specifically kitesurfing. Physical examination revealed a painful and swollen knee, with joint effusion and medial ecchymosis. He had a valgus laxity at both 0 and 30 degrees, a negative anterior drawer test, a positive posterior drawer test, and doubtful Lachman test.

**Fig. 1:**
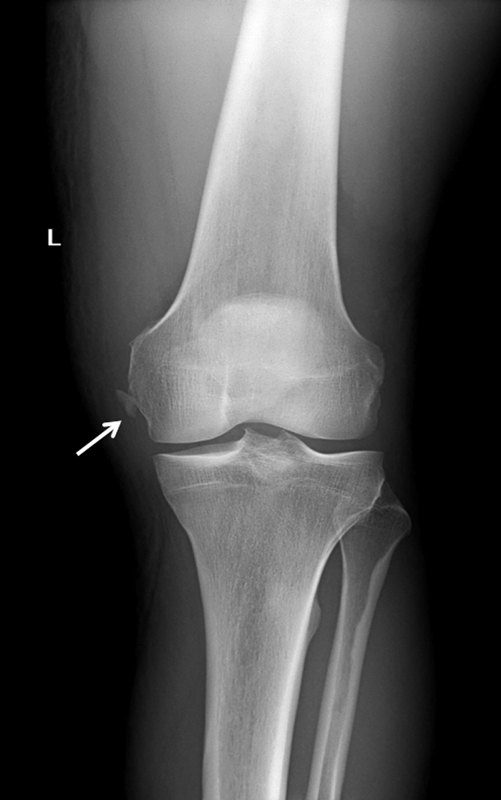
Anteroposterior X-ray of the right knee showing an avulsion fracture on the medial femoral condyle (arrow).

Anteroposterior and lateral X-rays of the knee were performed in the emergency room, showing an avulsion fracture on the medial femoral condyle ( Fig. 1 ). This finding, combined with the clinical presentation, pointed toward an avulsion of the MCL of the knee.

**Fig. 2:**
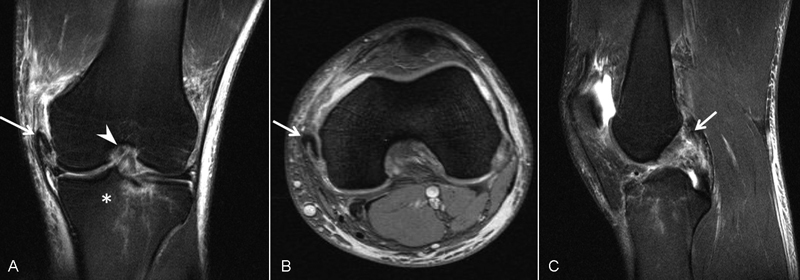
T2-weighted magnetic resonance imaging of the injured knee. ( **A** ) Coronal view: arrow, avulsion of the medial collateral ligament in the medial femoral condyle; asterisk, trabecular fractures with cortical collapse; arrowhead, altered imaging of the anterior cruciate ligament (keeping a normal route). ( **B** ) Axial view: arrow, avulsion of the medial collateral ligament in the medial femoral condyle. ( **C** ) Sagittal view: arrow, complete rupture of the posterior cruciate ligament.

**Fig. 3:**
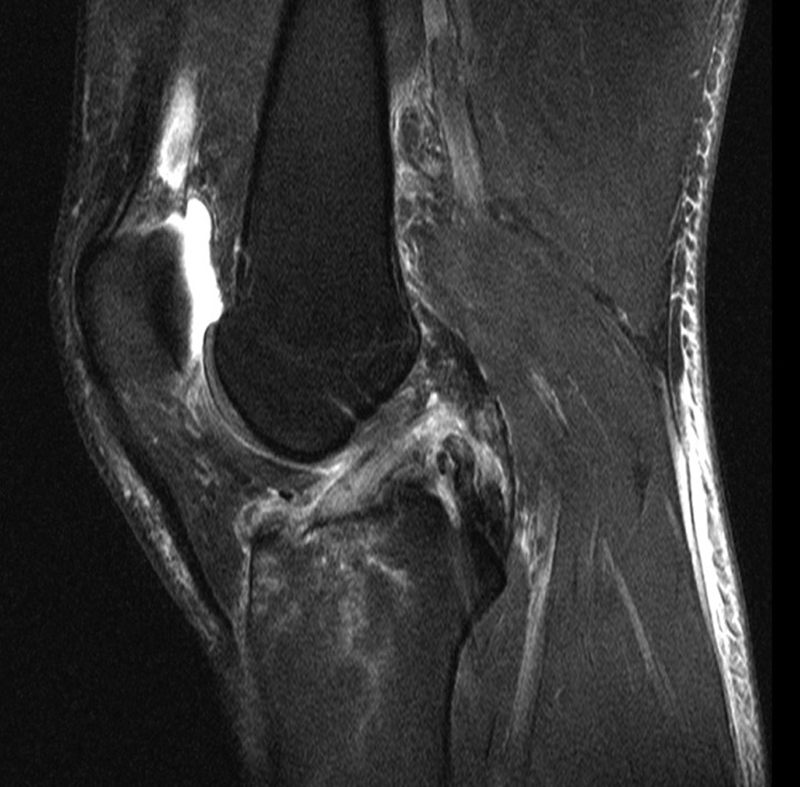
Magnetic resonance imaging of the injured knee showing the partial tear of the anterior cruciate ligament and the complete rupture of the posterior cruciate ligament.

The patient was discharged to our outpatients' clinics with protected weight-bearing using a brace and crutches. His magnetic resonance imaging (MRI) findings were as follows: avulsion of the MCL's origin in the medial femoral condyle with the bone fragment rotated and retracted together with the MCL, trabecular fractures adjacent to the posterolateral tibial spine with a minimum cortical collapse and a severe bone edema, altered imaging of the anterior cruciate ligament (ACL) keeping a normal route, which could be explained by a partial tear of the ligament, and complete rupture of the PCL with its proximal part retracted ( Figs. 2 and 3 ).

The patient was offered and consented for a navigated arthroscopic reconstruction of both ligaments.

The PCL was reconstructed using the double-bundle Achilles allograft technique, with an accessory posterolateral portal. 4 Within the same surgery, a medial femoral incision on the internal femoral condyle right on top of the anatomical insertion point of the MCL was performed observing the avulsion of the bone fragment rotated and distally retracted together with the MCL. A release and reinsertion with bone anchors and Spike Washer was performed.

Immediate physical therapy was prescribed, and passive range of motion was initiated. Progressive active therapy and weight-bearing were then initiated after 2 weeks.

**Fig. 4:**
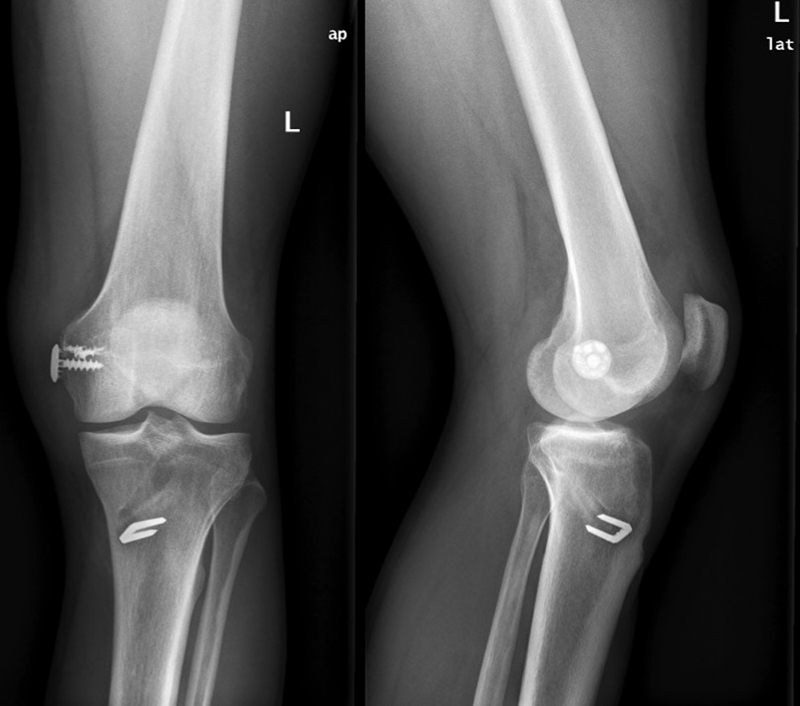
postoperative X-rays at the last follow-up.

**Fig. 5:**
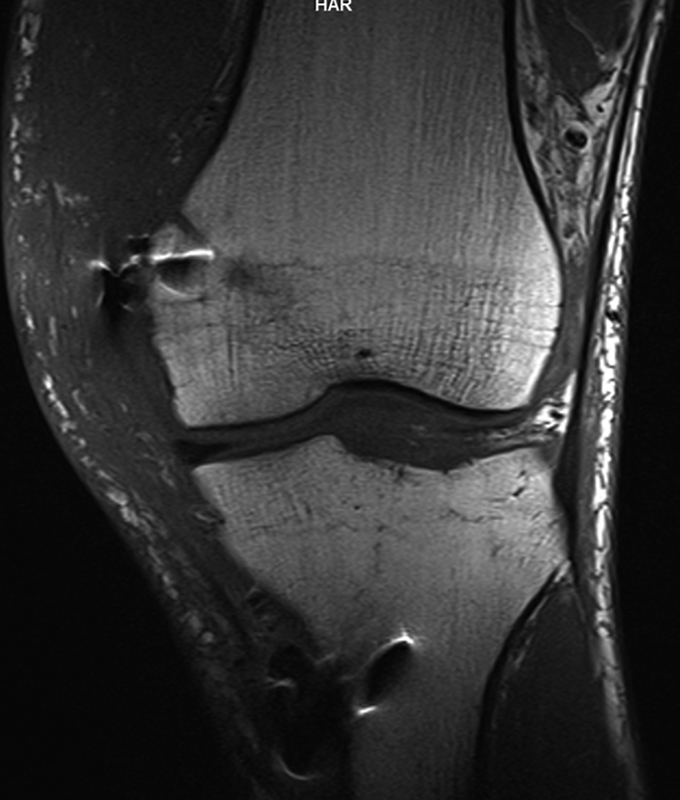
Magnetic resonance imaging showing the resolution of the bone edema.

At the last follow-up, 2 years after surgery, the patient enjoyed a 0/140-degree range of motion for flexion/extension ( Figs. 4 and 5 ). He had resumed his sports activity and was pain-free. At physical examination, no ligamentous instability could be detected. A new MRI was performed showing repair signs of the MCL without intraligamentous disruption.

## Discussion

MCL tear is the most common traumatic ligamentous injury of the knee in young adults and athletes. 1 5 Most isolated MCL injuries are treated nonoperatively, with patients achieving preinjury level. 6 7 However, MCL tear is frequently associated to other ligamentous or meniscal lesions of the knee, especially ACL. Whenever MCL injury is suspected, MRI is recommended to determine underlying ligamentous and meniscal injury that could need surgical repair. 8 Besides multiligamentous injuries, other indication for MCL repair is chronic instability. 7 9

Most MCL tears are due to disruption of fibers near the femoral insertion. 9 Very few avulsion injuries have been described in the adult population, and when a bone fragment is seen near the femoral insertion, the clinician traditionally considered a Pellegrini–Stieda's syndrome (PSD). The syndrome is defined as chronic knee pain together with a characteristic image in X-ray; a calcified formation in the region of the medial femoral condyle (at the MCL's origin). 10 Different etiologies have been considered for PSD. They mostly include a traumatic precedence, but chronic adductor tendinopathy, myositis ossificans, and idiopathic calcifications have also been described to cause PSD. A more recent study described a traumatic PSD in four patients. 11 All of them had a high-energy accident with complete PCL tear/avulsion and valgus instability but without MCL lesion upon MRI examination. Unlike our case, MRI findings described a periostic avulsion of the superior part of MCL with a distal retraction, and no bony avulsion could be seen. 11 Years after the initial traumatism, all four patients presented a painful calcification near the MCL insertion on the medial femoral condyle.

True bony avulsions of the MCL are extremely rare in the adult population, and a single article reports a MCL avulsion from both its proximal femoral and distal tibial attachments associated with subluxation of the medial meniscus. 12 This article, as such, is the first report of a bony avulsion of the proximal MCL associated with a PCL tear. Although combined MCL and PCL injury is a rare but well-recognized entity, in none of the reported cases, the MCL was not avulsed from its insertion but rather suffered from fiber disruption. 13 14

The proposed mechanism of injury in this case is a forceful valgus with external rotation associated with a hyperextension of the knee during the fall while practicing kitesurfing.

In conclusion, femoral avulsion of the MCL associated to PCL injury is a rare and previously nondescribed injury that, as opposed to most MCL isolated injuries, might benefit from early surgical reconstruction. When seen on a simple X-ray, as with Segond fracture, the clinician should have a high index of suspicion for a high-energy mechanism and a possible multiligamentous injury or other associated injuries. Therefore, an extensive physical examination should be done and further imaging (such as MRI) should be performed when facing this type of lesion. If diagnosis of the avulsion is confirmed or if a multiligamentous injury is detected, early surgical repair should be considered, including repairing the associated injuries.

## References

[1] Wijdicks C A Griffith C J Johansen S Engebretsen L LaPrade R F Injuries to the medial collateral ligament and associated medial structures of the knee J Bone Joint Surg Am 2010 92 05 1266 1280 20439679 10.2106/JBJS.I.01229

[2] Laprade R F Wijdicks C A The management of injuries to the medial side of the knee J Orthop Sports Phys Ther 2012 42 03 221 233 22382986 10.2519/jospt.2012.3624

[3] Dowd G S Reconstruction of the posterior cruciate ligament. Indications and results J Bone Joint Surg Br 2004 86 04 480 491 15174540

[4] Heinzelmann A D Barrett G R Posterior cruciate ligament reconstruction: Achilles tendon allograft, double bundle Clin Sports Med 2009 28 02 245 257, viii 19306733 10.1016/j.csm.2008.10.013

[5] Schein A Matcuk G Patel D Structure and function, injury, pathology, and treatment of the medial collateral ligament of the knee Emerg Radiol 2012 19 06 489 498 22890899 10.1007/s10140-012-1062-z

[6] Phisitkul P James S L Wolf B R Amendola A MCL injuries of the knee: current concepts review Iowa Orthop J 2006 26 77 90 16789454 PMC1888587

[7] Marchant M H Jr Tibor L M Sekiya J K Hardaker W T Jr Garrett W E Jr Taylor D C Management of medial-sided knee injuries, part 1: medial collateral ligament Am J Sports Med 2011 39 05 1102 1113 21148144 10.1177/0363546510385999

[8] Gottsegen C J Eyer B A White E A Learch T J Forrester D Avulsion fractures of the knee: imaging findings and clinical significance Radiographics 2008 28 06 1755 1770 18936034 10.1148/rg.286085503

[9] Jacobson K E Chi F S Evaluation and treatment of medial collateral ligament and medial-sided injuries of the knee Sports Med Arthrosc Rev 2006 14 02 58 66 17135948 10.1097/01.jsa.0000212305.47323.58

[10] Wang J C Shapiro M S Pellegrini-Stieda syndrome Am J Orthop 1995 24 06 493 497 7670873

[11] McAnally J L Southam S L Mlady G W New thoughts on the origin of Pellegrini-Stieda: the association of PCL injury and medial femoral epicondylar periosteal stripping Skeletal Radiol 2009 38 02 193 198 18985339 10.1007/s00256-008-0604-7

[12] Naik A M Rao S K Rao P S Medial collateral ligament avulsion from both tibial and femoral attachments: a case report J Orthop Surg (Hong Kong) 2007 15 01 78 80 17429123 10.1177/230949900701500117

[13] Bonadio M B Helito C P Foni N O Combined reconstruction of the posterior cruciate ligament and medial collateral ligament using a single femoral tunnel Knee Surg Sports Traumatol Arthrosc 2016 10.1007/s00167-016-4071-8 27000395

[14] Forsythe B Mascarenhas R Pombo M W Harner C D, eds.Combined injuries to the posterior cruciate ligament and medial collateral ligament of the knee Paris Springer 2012 421 426

